# A Case of Pleuritic Effusion

**Published:** 1881-06-20

**Authors:** Thomas F. Houston

**Affiliations:** Atlanta, Ga.


### A CASE OF PLEURITIC EFFUSION.

BY THOMAS F. HOUSTON, M. D., ATLANTA, GA.

During my recent visit to New York, I saw in the various hospitals visited, many cases of great interest, and one of them presented several features that I feel assured will interest the readers of this journal. In December, 1879, Mr. ———, a street car conductor, presented himself to Dr. Kotzenbach, giving a history of an attack of pleurisy on the right side. The patient was very weak, could not walk more than a block at a time. Physical examination showed the left lung sound, but overworked. The right side gave complete flatness on percussion, vocal resonance absent, vocal fremitus slightly increased, and no perceptible respiratory murmur. The heart was displaced into the left axillary region, the patient could feel its pulsations against his left arm. Dr. K. inserted a hypodermic needle under the eighth rib, and filled the syringe with pus. Using Flint's trocar, he drew off one hundred and thirty-eight ounces of yellowish creamy sero-pus. Mr. ——— immediately declared that he felt very much better, and improved so rapidly that on the third day he walked several miles, and continued his vocation in apparent good health for ten months. He then returned, complaining of the same symptoms very much aggravated. Dr. Kotzenbach again aspirated him; this time the effusion amounted to one hundred and fifty-eight ounces. I saw him six months later. There was slight cardiac displacement an inch or so to the left; the percussion note over the affected side was absolutely wooden in character, due to the induration and thickening of the pleura, absence of respiratory murmur and vocal resonance; slight increase of vocal fremitus. Again using the hypodermic needle as an exploring trocar, we found an effusion of greenish-yellow sero-pus as high as the seventh interspace. Not having the aspirator at hand, Dr. K. directed him to continue his work until his symptoms again became severe, and then to report for aspiration. The points of interest that present themselves to me are—the entire absence of all cardiac lesions, other than slight palpitation, which disappeared when the fluid was withdrawn. The large amount of the effusion and the fact that it caused absolutely no constitutional symptoms save weakness and anorexia. There was not a trace of pyrexia, the themometer never reaching 100°F. At first glance it would seem almost impossible for so large a quantity of sero-pus to be months retained in the pleural cavity without its being absorbed and producing septicæmia with all its terrible consequences. The only way that I can account for this patient's exemption from the usual course of the disease, is that structural changes had taken place in the pleura itself, together with the thick layer of fibrin that had been poured out on the surface in contact with the fluid, thus acting as a mechanical impediment to its absorption by the sub-pleural vessels. The longer that this fluid remains the thicker grows the deposit of fibrin, until the pleura was indurated and hypertrophied to such an extent that its percussion note was so flat that it masked any vesicular quality that might have remained in the lung beneath. The great quantity was also another means of preventing the absorption. Its pressure on the lung obstructed if not occluded the pulmonary circulation, and the sub-pleural veins thus causing capillary engorgement. And the bulging into the intercostal spaces also interfered with the venous circulation found there. This stasis of the circulation would have a tendency rather to increase than to diminish its bulk by the transudation of the watery products of the blood. In fact, I think this had much to do with the amount of the fluid, for where a serous or even any other membrane had become structurally changed to the extent of an entire loss of its power of absorption, its power of excretion also must have been seriously impaired if not entirely destroyed. I have not had time to carefully investigate the literature of the subject, but I do not remember to have seen a parallel case reported, either in the amount of the fluid or the lack of constitutional disturbance.

